# Management and outcome of displaced acetabular fractures in the oldest-old: a retrospective analysis of orif and acute total hip arthroplasty

**DOI:** 10.1007/s00402-026-06511-0

**Published:** 2026-09-18

**Authors:** Ioannis Mamoutakis, Klemens Horst, Hatem Alabdulrahman, Rald V. M. Groven, Felix M. Bläsius, Frank Hildebrand, Till Berk, Eftychios Bolierakis

**Affiliations:** 1 Department of Orthopedics, Trauma, and Reconstructive Surgery, University Hospital RWTH Aachen, Aachen, Germany; 2 Department of Trauma, Hand- and Reconstructive Surgery, University Hospital Münster, Münster, Germany

**Keywords:** Geriatric acetabular fracture, Osteosynthesis, Arthroplasty, Endoprosthesis, Remobilization

## Abstract

**Purpose:**

The optimal surgical management of geriatric acetabular fractures remains controversial. This study compared short-term outcomes following open reduction and internal fixation (ORIF) or acute total hip arthroplasty (THA) with or without additional osteosynthesis (THA ± ORIF) and evaluated the impact of a new treatment algorithm (standard operative procedure, SOP) that favors the use of THA in elderly and medically comorbid patients.

**Methods:**

In this retrospective cohort study, patients aged ≥ 70 years with isolated trauma-associated unilateral displaced acetabular fractures who underwent surgical treatment at a single Level I trauma center between January 2016–March 2019 (old SOP) and January 2022–December 2025 (new SOP) were included. Patients underwent either ORIF or THA ± ORIF. The transitional implementation phase of the new SOP was excluded. The primary outcome was postoperative time to gait mobilization. Secondary outcomes included pain at discharge, functional mobility, complications, mortality, quality of fracture reduction, and conversion to THA following initial ORIF. Because treatment allocation followed different institutional algorithms across the study periods, the study primarily evaluates an evolving treatment strategy rather than directly comparing ORIF and THA.

**Results:**

Sixty patients (median age 82 years, IQR 78–87; median Charlson Comorbidity Index 5, IQR 4–7) were included. The most common fracture pattern according to the Letournel classification was the anterior column with posterior hemitransverse fracture (*n* = 35). Thirty-five patients underwent ORIF and 25 underwent THA ± ORIF. Compared with ORIF, THA ± ORIF was associated with significantly faster remobilization (median 3 vs. 6 days, IQR 2–3 vs. 4–7; *p* < 0.05), lower pain levels at discharge (VAS 1–3: 96% vs. 37.1%; *p* < 0.05), and superior functional mobility at discharge. Complication rates, readmissions, length of stay, and in-hospital mortality did not differ significantly between groups. These findings remained consistent after implementation and refinement of the newly introduced SOP despite the increasing use of THA ± ORIF in older and more comorbid patients.

**Conclusion:**

A structured treatment algorithm incorporating THA ± ORIF was associated with earlier remobilization and lower pain levels at discharge without increasing complication rates compared with ORIF in geriatric patients with displaced acetabular fractures. These findings support differentiated treatment allocation and the safe use of acute THA in carefully selected elderly patients.

**Supplementary Information:**

The online version contains supplementary material available at https://doi.org/10.1007/s00402-026-06511-0.

## Introduction

A sharp increase in geriatric acetabular fractures has been observed in recent decades due to the aging global population, with projections suggesting a 1.7-fold rise by 2030 [1–3]. These fractures predominantly affect older male patients, accounting for approximately 62% of cases, and are mostly caused by low-energy trauma [4]. Mortality within the first year after a geriatric acetabular fracture has been reported to reach 44% in non-operatively treated patients compared with approximately 12% in surgically treated acetabular fractures [5].

The surgical management of geriatric acetabular fractures remains controversial, with no universally accepted gold standard [6]. Surgical strategies for displaced fractures include open reduction and internal fixation (ORIF) and total hip arthroplasty (THA), with or without additional fixation [7]. Overall, ORIF remains the gold standard for acetabular fracture treatment, as it allows anatomical fracture reduction and preservation of the native hip joint. However, in elderly patients the procedure can be technically demanding due to poor bone quality, complex fracture patterns, and the limited ability of some patients to comply with postoperative weight-bearing restrictions [6, 8–10]. In contrast, THA, alone or combined with additional fixation, has shown promising results in selected geriatric patients by enabling earlier mobilization and improved functional recovery. However, it may also be associated with increased complications, due to the higher surgical burden or procedure-specific risks, such as periprosthetic infection and dislocation [6, 11–15].

One critical aspect in the current literature on this particular topic is the definition of geriatric trauma patients, in which most studies defined this group as patients aged 60 years or older [6, 7, 13, 15]. Thus, there is limited literature on septua- and octogenarian patient cohorts with substantial comorbidity and frailty. The present study, therefore, specifically focuses on this demographic group.

In 2019, our institution introduced a revised standardized operative procedure (SOP) for the management of geriatric acetabular fractures following the implementation of a cementless modular revision cup system. In a previous study, this strategy was associated with favourable clinical outcomes in patients aged > 55 years [16]. However, interpretation of those findings was limited because the study included the early implementation phase of both the revised SOP and the implant concept, a period potentially influenced by a surgical learning curve and evolving patient selection. Moreover, the inclusion of patients younger than 70 years may have contributed to the favourable outcomes. These limitations provided the rationale for the present study, which focuses on patients aged ≥ 70 years after completion of the SOP implementation phase.

Thus, the present cohort study retrospectively evaluates short-term outcomes after ORIF and THA with or without additional osteosynthesis (THA ± ORIF) in geriatric patients with displaced acetabular fractures, with a particular focus on older (≥ 70 years) and medically frail patients. The current study assesses the effectiveness of a refined institutional SOP intended to facilitate the use of acute THA in the aforementioned patient group when clinically indicated. By comparing historical patient cohorts treated before and after establishment of the SOP, the study further aims to evaluate the impact and consistency of this treatment strategy in routine clinical practice. We hypothesized that the standardized use of THA with or without additional osteosynthesis in this vulnerable patient population would facilitate early remobilization without increasing complication rates.

## Methods

### Study design and ethical considerations

The study was approved by the local Ethics Committee. Informed consent for data collection was obtained from all patients during hospitalization. The study was conducted in accordance with the Declaration of Helsinki in its most recent form and Good Clinical Practice Guidelines, and is reported in accordance with the Strengthening the Reporting of Observational Studies in Epidemiology (STROBE) guidelines [17].

### Study population

Patients aged ≥ 70 years who underwent surgical treatment for displaced acetabular fractures at an academic Level 1 trauma center were included. The study period comprised two distinct treatment periods. The first period ranged from January 2016 to March 2019, while the second period ranged from January 2022 to December 2025. In April 2019, a new SOP for the management of geriatric acetabular fractures was introduced, that facilitates the conceptual use of acute THA in elderly and medically comorbid patients when clinically indicated [16]. Concurrently, the primary implant used for THA was changed from various cementless modular revision cups without iliac peg to a cementless modular revision cup with iliac peg (MRS-Titan^®^ Maximum, Peter Brehm GmbH, Weisendorf, Germany). The period from April 2019 to December 2021 was excluded from analysis because the introduction and validation phase of the revised treatment protocol and the new implant were expected to introduce variability in patient selection, treatment strategies, and clinical outcomes. In both treatment periods, surgical decision-making was primarily guided by fracture morphology and patient-related factors, informed by previously published predictors of outcome and early failure following acetabular fracture fixation [18], including roof (dome) impaction, femoral head injury, hip dislocation, multifragmentary posterior wall involvement, pre-existing osteoarthritis, and overall fracture characteristics. However, prior to April 2019, the indication for acute total hip arthroplasty in older and medically frail patients was applied more cautiously due to the limitations of the available endoprosthetic implant systems. Following validation of the revised treatment protocol, treatment decisions were made according to the newly introduced standardized institutional strategy.

The revised SOP is summarized in Supplementary Figure S1 [16]. Briefly, treatment selection was based on a structured assessment of both fracture-related and patient-related factors. Initial evaluation considered fracture displacement and the presence of pre-existing hip osteoarthritis. In displaced fractures, additional radiographic characteristics including dome impaction, associated femoral head or neck fractures, fracture reducibility, supra-acetabular bone stock, roof arc angle, and quadrilateral plate involvement were evaluated to determine whether joint-preserving ORIF was feasible or whether acute THA ± ORIF was preferred. Patients selected for acute THA underwent either stand-alone THA or THA + ORIF according to the remaining acetabular stability. In general, stand-alone THA was performed when the supraacetabular corridor remained intact, the roof arc angle exceeded 60°, and only infratectal quadrilateral plate involvement was present; otherwise, THA was combined with ORIF. Patient-specific characteristics, including age-adjusted Charlson Comorbidity Index, neurological or psychiatric impairment, and the anticipated ability to comply with postoperative partial weight-bearing restrictions, were incorporated into the decision-making process. The SOP was intended to standardize treatment allocation while allowing individualized surgical decision-making based on the combined assessment of fracture morphology and patient characteristics [16].

Importantly, treatment allocation was not randomized. Following implementation of the revised SOP, older and medically frail patients were considered more frequently for acute THA when fracture morphology and patient characteristics suggested that joint-preserving fixation would be less favourable. Therefore, treatment allocation was inherently influenced by patient characteristics and represents routine clinical decision-making.

**Fig. 1:**
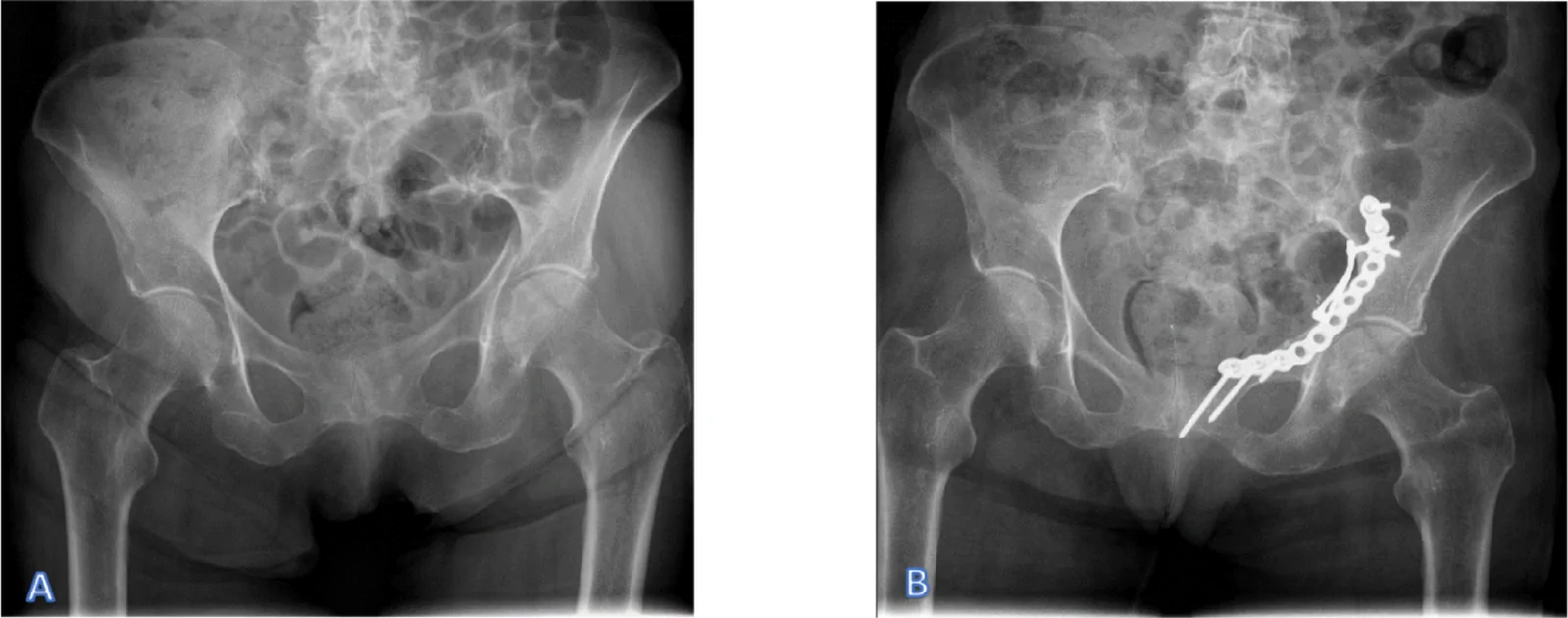
X-ray images extracted from the hospital’s radiology system (Philips IntelliSpace PACS) representing the ORIF treatment: **A** Preoperative x-ray imaging; **B** Postoperative x-ray imaging after ORIF

**Fig. 2:**
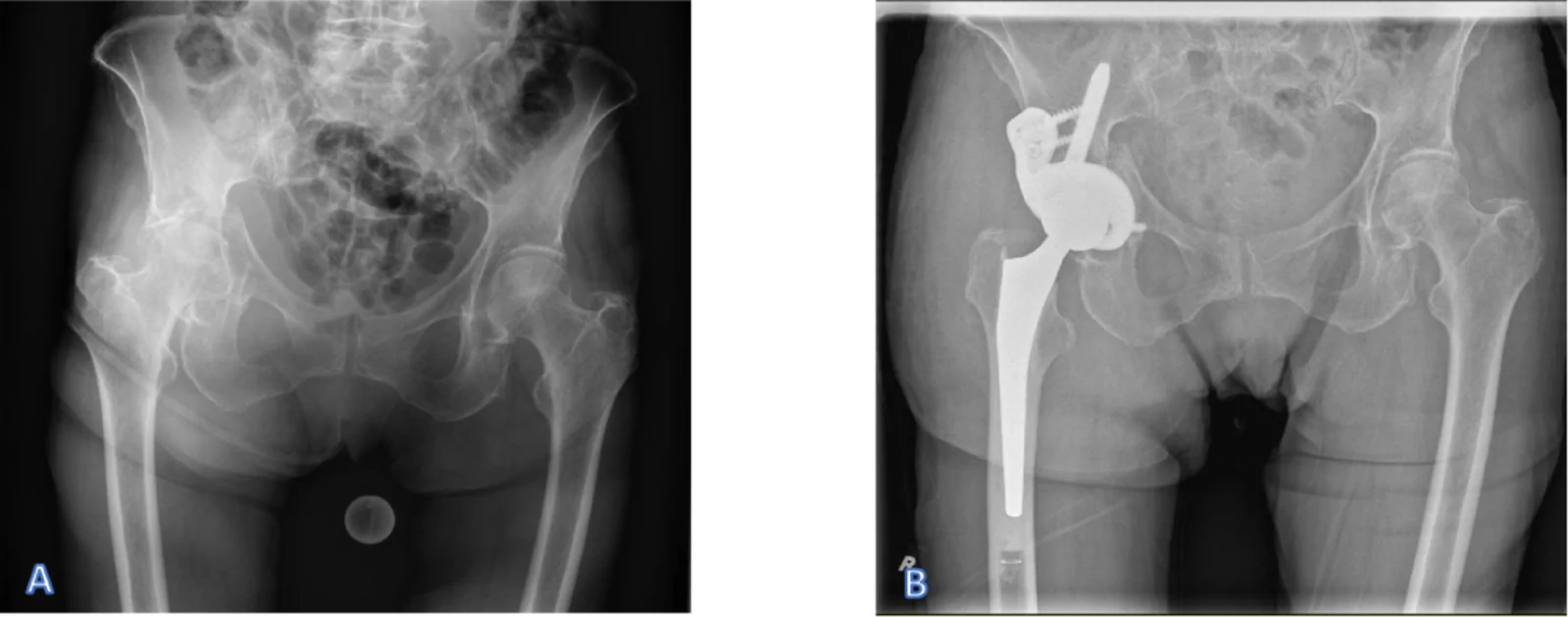
X-ray images extracted from the hospital’s radiology system (Philips IntelliSpace PACS) representing the stand-alone THA treatment: **A** Preoperative x-ray imaging; **B** Postoperative x-ray imaging after stand-alone THA

**Fig. 3:**
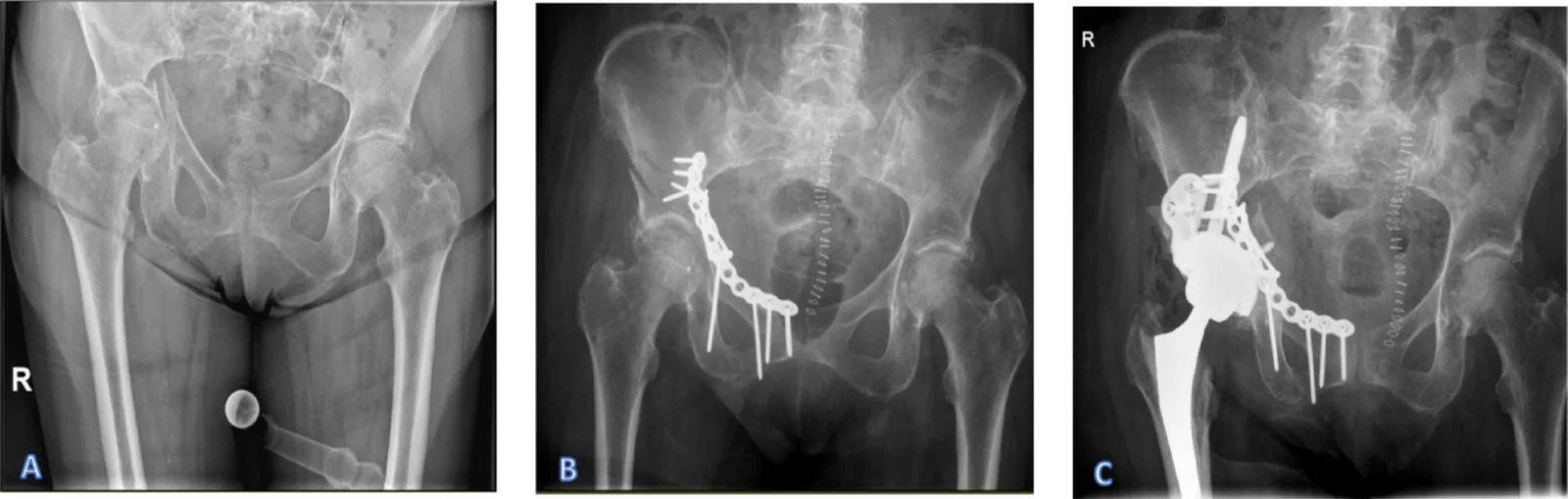
X-ray images extracted from the hospital’s radiology system (Philips IntelliSpace PACS) representing the THA + ORIF treatment: **A** Preoperative x-ray imaging; **B** Postoperative x-ray imaging after ORIF; **C** Postoperative x-ray imaging after THA + ORIF

Fractures were considered displaced if there was a step-off or gap of the articular surface greater than 2 mm [19]. Eligible cases were identified through retrospective screening of administrative patient data using the International Classification of Diseases (ICD). Patients were excluded if they had bilateral acetabular fractures, pathological fractures, periprosthetic fractures, multiple injuries, or additional concomitant pelvic ring injuries. The study population was primarily divided according to the performed surgical procedure performed into two treatment groups: ORIF and THA with or without additional osteosynthesis (THA ± ORIF) (Figs. 1, 2 and 3).

Postoperative rehabilitation protocols differed according to the surgical procedure. Patients treated with THA ± ORIF were mobilised with full weight bearing as tolerated, although walking aids were generally recommended during the first six postoperative weeks. Patients treated with ORIF were advised to adhere to partial weight bearing (up to 15 kg) using walking aids for the first eight postoperative weeks, followed by a gradual progression to full weight bearing as tolerated [16].

### Outcome measures and data collection

The primary outcome of this study was the postoperative time interval (days) to remobilization into gait, defined as physiotherapy-assisted walking for at least 20 m. This outcome was routinely documented by treating physiotherapists in the hospital’s electronic medical record as part of standardized inpatient rehabilitation and was retrospectively extracted for the present analysis. Secondary outcomes included perioperative general and surgery related complication rates, in-hospital mortality, radiological outcomes (postoperative step-off and gap measurements for ORIF), length of hospital stay, functional independence upon discharge, and postoperative pain levels.

The following data were extracted: age, gender, hospital length of hospital stay (LOS), length of intensive care unit (ICU) stay, American Society of Anesthesiologists (ASA) classification, age-adjusted Charlson Comorbidity Index, pre-injury living situation and mobility status, fracture classification (Judet and Letournel classification [20]), procedure type, preoperative and postoperative fracture gap and step-off measurements, time interval (days) to remobilization into assisted walking, functional mobility at discharge, place of discharge and pain levels upon discharge (Visual Analogue Scale), general and implant-related complications, readmission rates, and rates of conversion to THA following initial ORIF.

### Statistical analysis

Statistical analysis and was performed using IBM SPSS Statistics for Windows (Version 28.0, IBM Corp; Armonk, NY). The normality of continuous variables was assessed using Q-Q plots and the Shapiro-Wilk test. As all continuous variables were non-normally distributed, continuous data were summarized using median and interquartile range (IQR). Categorical variables are presented as counts and percentages. Between-group comparisons were conducted using the Mann-Whitney U test for continuous variables and the chi-square test for categorical variables. Statistical significance was defined as *p* < 0.05.

## Results

### Demographic and injury characteristics

A total of 60 patients were included, with a median age of 82 years (IQR 78–87), median Charlson Comorbidity Index of 5 (IQR 4–7), and median ASA score of 3 (IQR 2–3). ORIF was performed in 35 patients (58.3%), while 25 (41.7%) underwent THA ± ORIF, of whom 16 (64%) received THA + ORIF and 9 (36%) were treated with stand-alone THA. Most patients were male (*n* = 36, 60%), and the predominant fracture pattern was anterior column with posterior hemi-transverse fracture (*n* = 35, 58.3%). Prior to injury, most patients lived at home, independently or with assistance, and were ambulatory without aids or with minimal support (Table 1).

Baseline demographic and injury characteristics did not differ significantly between the 2016–2019 and 2022–2025 treatment periods. However, THA ± ORIF was more frequently performed in 2022–2025 than in 2016–2019 (*n* = 19 vs. *n* = 6) (Tables 1 and 2). No significant baseline differences were observed between treatment cohorts within the 2016–2019 treatment period. In contrast, in the 2022–2025 interval, patients treated with THA ± ORIF were significantly older (median 87 vs. 77 years, IQR 82–88 vs. 75–80; *p* < 0.05) and had higher Charlson Comorbidity Index scores (median 6 vs. 4, IQR 5–7 vs. 3–6; *p* < 0.05) than those treated with ORIF (Tables 1 and 2).

**Table 1:** Baseline demographic and injury characteristics stratified by treatment period

	Overall (*n* = 60)	2016–2019 (*n* = 28)	2022–2025 (*n* = 32)	*P*-value
Age in years, median (IQR)	82 (78–87)	82 (78–88)	83 (77–87)	≥ 0.05
*Gender, n (%)*				
Female	24 (40.0)	10 (35.7)	14 (43.7)	≥ 0.05
Charlson comorbidity index, median (IQR)	5 (4–7)	5 (4–7)	5 (4–7)	≥ 0.05
ASA score, median (IQR)	3 (2–3)	3 (2–3)	3 (2–3)	≥ 0.05
*Living situation prior to fracture, n (%)*				
At home, independent	31 (51.7)	11 (39.3)	20 (62.5)	≥ 0.05
At home, care-dependent	21 (35)	12 (42.9)	9 (28.1)	
At nursing home, independent	2 (3.3)	1 (3.6)	1 (3.1)	
At nursing home, care dependent	6 (10)	4 (14.3)	2 (6.3)	
*Pre-fracture mobility, n (%)*				
Independent (no walking aid)	29 (48.3)	14 (50)	15 (46.9)	≥ 0.05
Cane/ single crutch	5 (8.3)	2 (7.1)	3 (9.4)	
Two crutches	0	0	0	
Walker/ rollator	22 (36.7)	10 (35.7)	12 (37.5)	
Wheelchair	1 (1.7)	0	1 (3.1)	
*Judet/Letournel fracture classification n (%)*				
Elementary fracture patterns				≥ 0.05
* PW*	6 (10)	2 (7.1)	4 (12.5)	
* PC*	0	0	0	
* AW*	0	0	0	
* AC*	9 (15)	3 (10.7)	6 (12.5)	
* Transverse*	2 (3.3)	1 (3.6)	1 (3.1)	
Associated fracture patterns				
* T-shaped*	1 (1.7)	1 (3.6)	0	
* PC with PW*	0	0	0	
* Transverse with PW*	0	0	0	
* AC with PHT*	35 (58.3)	18 (64.3)	17 (53.1)	
* ABC*	7 (11.7)	3 (10.7)	4 (12.5)	
Preoperative fracture gap in mm, median (IQR)	8 (5–14)	8 (6–10)	10 (5–14)	≥ 0.05
Preoperative fracture step in mm, median (IQR)	4 (3–8)	3 (3–5)	5 (3–13)	≥ 0.05

*ORIF* open reduction and internal fixation, *THA* total hip arthroplasty, *n* number, *IQR* interquartile range, *ASA* American society of anesthesiologists, *PW* posterior wall, *PC* posterior column, *AW* anterior wall, *AC* anterior column, *PHT* posterior hemitransverse, *ABC* associated both column

**Table 2:** Baseline demographic and injury characteristics stratified by treatment group within treatment period

	ORIF 2016–2019 (*n* = 22)	ORIF 2022–2025 (*n* = 13)	THA ± ORIF 2016–2019 (*n* = 6)	THA ± ORIF 2022–2025 (*n* = 19)
Age in years, median (IQR)	82 (78–87)	77 (75–80)	83 (72–91)	87 (83–88)
*Gender, n (%)*				
Female	6 (27.3)	6 (46.1)	4 (66.7)	8 (42.1)
Charlson comorbidity index, median (IQR)	6 (5–7)	4 (3–6)	5 (3–5)	6 (5–7)
ASA score, median (IQR)	3 (2–3)	3 (2–3)	3 (2–3)	3 (2–3)
*Living situation prior to fracture, n (%)*				
At home, independent	8 (36.4)	10 (76.9)	3 (50)	10 (52.6)
At home, care-dependent	10 (45.5)	2 (15.4)	92(33.3)	7 (36.8)
At nursing home, independent	1 (4.5)	1 (7.7)	0	0
At nursing home, care dependent	3 (13.6)	0	1 (16.7)	2 (10.5)
*Pre-fracture mobility, n (%)*				
Independent (no walking aid)	10 (45.5)	10 (76.9)	4 (66.7)	5 (26.3)
Cane/ single crutch	1 (4.5)	0	1 (16.7)	3 (15.8)
Two crutches	0	0	0	0
Walker/ rollator	10 (45.5)	2 (15.4	0	10 (52.6)
Wheelchair	0	1 (7.7)	0	0
*Judet/Letournel fracture classification n (%)*				
Elementary fracture patterns				
* PW*	1 (4.5)	2 (15.4)	1 (16.7)	2 (10.5)
* PC*	0	0	0	0
* AW*	0	0	0	0
* AC*	3 (14.6)	4 (30.8)	0	2 (10.5)
* Transverse*	1 (34.5)	0	0	1 (5.3)
Associated fracture patterns				
* T-shaped*	1 (4.5)	0	0	0
* PC with PW*	0	0	0	0
* Transverse with PW*	0	0	0	0
* AC with PHT*	13 (59.1)	7 (53.8)	5 (83.3)	10 (52.6)
* ABC*	3 (13.6)	0	0	4 (21.1)
Preoperative fracture gap in mm, median (IQR)	8 (5–10)	8 (5–13)	11 (8–19)	10 (5–15)
Preoperative fracture step in mm, median (IQR)	3 (3–5)	5 (3–11)	3 (3–6)	5 (4–14)

*ORIF* open reduction and internal fixation, *THA* total hip arthroplasty, *n* number, *IQR* interquartile range, *ASA* American society of anesthesiologists, *PW* posterior wall, *PC* posterior column, *AW* anterior wall, *AC* anterior column, *PHT* posterior hemitransverse, *ABC* associated both column

### Course of in-hospital treatment with clinical and radiological outcomes

Across the entire study cohort, median time to surgery showed no significant differences between the treatment cohorts. Compared to ORIF, THA ± ORIF was associated with significantly lower pain levels at discharge (VAS 1–3: 96% vs. 37.1%; *p* < 0.05), earlier remobilization into assisted walking (median 3 vs. 6 days, IQR 2–3 vs. 4–7; *p* < 0.05), and superior mobility at discharge, with all THA ± ORIF patients achieving independent walking with assistive devices compared to 17 (48.6%) in the ORIF cohort (*p* < 0.05). Length of hospitalization was comparable between groups (median 14 days; *p* ≥ 0.05). In the ORIF cohort, postoperative CT showed satisfactory reduction with a median fracture gap of 2 mm (IQR 2–5) and fracture step size of 2 mm (IQR 1–3)

The most common complications across the entire study cohort were pneumonia (*n* = 9; 15%) and urinary tract infections (*n* = 7; 11.7%). Implant-related complications included postoperative deep tissue infection (*n* = 3; 5%), intraoperative bleeding (*n* = 2; 3.3%), postoperative hematoma/seroma formation (*n* = 2; 3.3%), implant loosening (*n* = 2; 3.3%), and THA dislocation (*n* = 1; 1.7%). Overall complication and readmission rates did not differ significantly between the cohorts. In the ORIF cohort, three patients (8.6%) underwent secondary conversion to THA after a median time interval of 319 days. The in-hospital mortality rate was 3.3% (*n* = 2), with both deaths occurring in the ORIF cohort (Tables 3 and 4).

After stratifying by treatment period, the clinical outcome patterns between the treatment cohorts remained largely comparable. However, within the 2022–2025 treatment period, patients treated with THA ± ORIF reported significantly lower pain levels at discharge, with a higher proportion reporting mild pain (VAS 1–3; *n* = 10 vs. *n* = 4, *p* < 0.05) (Table 3). Comparison between treatment periods, irrespective of surgical treatment principle, demonstrated largely comparable clinical and radiological outcomes. Nevertheless, patients treated in the 2022–2025 treatment period achieved significantly earlier remobilization into walking and superior mobility at discharge compared to those treated in 2016–2019, with 27 patients (84.4%) achieving independent walking with assistive devices versus 13 patients (46.4%) in 2016–2019 (*p* < 0.05) (Table 5).

**Table 3:** Mortality and complications stratified by treatment period

	Overall (*n* = 60)	2016–2019 (*n* = 28)	2022–2025 (*n* = 32)	*P*-value
Mortality, *n* (%)	2 (3.3)	1 (3.6)	1 (3.1)	≥ 0.05
Pneumonia, *n* (%)	9 (15)	5 (17.8)	4 (12.5)	≥ 0.05
Urinary tract infection, *n* (%)	7 (11.7)	5 (17.8)	2 (6.2)	≥ 0.05
Thrombosis, *n* (%)	1 (1.7)	1 (3.6)	0	≥ 0.05
Pulmonary embolism, *n* (%)	1 (2.4)	1 (3.6)	0	≥ 0.05
ARDS, *n* (%)	3 (5)	0	3 (9.4)	≥ 0.05
MOF, *n* (%)	4 (6.7)	0	4 (12.5)	≥ 0.05
Neurological deficits, *n* (%)	1 (1.7)	0	1 (3.1)	≥ 0.05
Deep tissue infection, *n* (%)	3 (5)	1(3.6)	2(6.2)	≥ 0.05
Hematoma/seroma formation, *n* (%)	2 (3.3)	0	2 (6.3)	≥ 0.05
Intraoperative bleeding, *n* (%)	2 (3.3)	1 (3.6)	1 (3.1)	≥ 0.05
Implant loosening, *n* (%)	2 (3.3)	1 (3.6)	1 (3.1)	≥ 0.05
THA dislocation, *n* (%)	1 (1.7)	1 (3.6)	0	≥ 0.05
Readmission, *n* (%)	6 (10)	4 (14.3)	2(6.2)	≥ 0.05
Conversion to THA, *n *(%)	3 (5)	2 (7.1)	1 (3.1)	≥ 0.05

*ORIF* open reduction and internal fixation, *THA* total hip arthroplasty, *n* number, *ARDS* acute respiratory distress syndrome, *MOF* multiple organ failure

**Table 4:** Mortality and Complications stratified by treatment cohort within treatment period

	ORIF 2016–2019 (*n* = 22)	ORIF 2022–2025 (*n* = 13)	THA ± ORIF 2016–2019 (*n* = 6)	THA ± ORIF 2022–2025 (*n* = 19)
Mortality, *n* (%)	1 (4.5)	1 (7.7)	0	0
Pneumonia, *n* (%)	4 (18.2)	3 (23.1)	1 (16.7)	1 (5.3)
Urinary tract infection, *n* (%)	4 (18.2)	0	1 (16.7)	2 (10.5)
Thrombosis, *n* (%)	1 (4.5)	0	0	0
Pulmonary embolism, *n* (%)	1 (4.5%)	0	0	0
ARDS, *n* (%)	0	2 (15.4)	0	1 (5.3)
MOF, *n* (%)	0	1 (7.7)	0	3 (15.8)
Neurological deficits, *n* (%)	0	0	0	1 (5.3)
Deep tissue infection, *n* (%)	1 (4.5)	1 (7.7)	0	1 (5.3)
Hematoma/seroma formation, *n* (%)	0	1 (7.7)	0	1 (5.3)
Intraoperative bleeding, *n* (%)	1 (4.5)	0	0	1 (5.3)
Implant loosening, *n* (%)	1 (4.5)	1 (7.7)	0	0
THA dislocation, *n* (%)	–	–	1 (16.7)	0
Readmission, *n* (%)	3 (13.6)	2 (15.4)	1 (16.7)	0
Conversion to THA, *n* (%)	2 (9.1)	1 (7.7)	–	–

*ORIF* open reduction and internal fixation, *THA* total hip arthroplasty, *n* number, *ARDS* acute respiratory distress syndrome, *MOF* multiple organ failure

**Table 5:** Clinical and radiological outcomes stratified by treatment cohort within treatment period

	ORIF 2016–2019 (*n* = 22)	ORIF 2022–2025 (*n* = 13)	THA ± ORIF 2016–2019 (*n* = 6)	THA ± ORIF 2022–2025 (*n* = 19)
Postoperative fracture gap in mm, median (IQR)	2(2–4)	4(2–4)	–	–
Postoperative fracture step in mm, median (IQR)	2 (1–3)	3(2–4)	–	–
Time to surgery in days, median (IQR)	5 (4–5)	6 (5–7)	5 (4–5)	5 (4–5)
Time interval in days from operation to mobilization into walking, median (IQR)	6 (4–7)	3 (3–6)	3 (2–3)	2 (1–2)
LOS ICU in days, median (IQR)	0 (0–3)	0 (0–1)	0 (0–1)	1 (0–2)
LOS in days, median (IQR)	14 (11–17)	14 (12–17)	13 (10–27)	11 (10–15)
*Mobility at discharge, n (%)*				
Independent (no walking aid)	0	0	0	0
Cane/ single crutch	0	1 (7.7)	0	0
Two crutches	1 (4.5)	1 (7.7)	1 (16.7)	5 (26.3)
Walker/ rollator	7 (31.8)	7 (53.8)	5 (83.3)	14 (73.7)
Wheelchair	12 (54.5)	2 (15.4)	0	0
Non-ambulatory	2 (9.1)	2 (15.4)	0	0
*Discharge destination, n (%)*				
Home	3 (13.6)	4 (30.8)	0	2 (10.5)
Nursing home	5 (22.7)	2 (15.4)	1 (16.7)	2(10.5)
Acute geriatric care unit	8 (36.4)	2 (15.4)	2 (33.3)	8 (42.1)
Rehabilitation clinic	1 (4.5)	0	1 (16.7)	0
Other Hospital	5 (2.7)	4 (30.8)	2 (33.3)	2 (10.5)
Other	0	1 (7.7)	0	5 (26.3)
*Pain levels on discharge*				
No pain, VAS 0	1 (4.5)	0	0	5 (26.3)
Mild pain, VAS 1–3	10 (45.5)	2 (15.4)	5 (83.3)	14 (73.7)
Moderate pain, VAS 4–6	9 (40.9)	9 (69.2)	1 (16.7)	0
Severe pain, VAS 7–10	2 (9.1)	2 (15.4)	0	0

*ORIF* open reduction and internal fixation,*THA* total hip arthroplasty, *n* number, IQR interquartile range, *ASA* American society of anesthesiologists, *VAS* visual analog scale

## Discussion

This study evaluated short-term clinical and radiological outcomes during index hospitalization in geriatric patients aged ≥ 70 years with displaced acetabular fractures treated with either ORIF or THA ± ORIF. Building upon the implementation and subsequent refinement of our institutional SOP designed to facilitate the use of acute THA in elderly and medically comorbid patients in indicated cases, the present analyses sought to determine whether this treatment strategy could efficiently improve early postoperative recovery without increasing treatment-related risks. Thus, the study primarily reflects the performance of a structured treatment algorithm in routine clinical practice rather than an absolute direct comparison of ORIF and THA. In line with our hypothesis, the main study findings were as follows:

1. THA ± ORIF was associated with a significantly shorter time to postoperative gait mobilization.
2. Patients treated with THA ± ORIF reported lower pain levels at discharge and demonstrated improved functional mobility.
3. No significant differences were observed in short-term mortality or complication rates between treatment strategies.
4. Comparing the two distinct treatment periods, the more frequent use of THA ± ORIF in the 2022–2025 period was associated with faster re-mobilization and lower pain levels.

The demographic characteristics of the present cohort are consistent with previously reported data on geriatric acetabular fractures, which describe a predominance of low-energy trauma mechanisms and a substantial burden of comorbidities [5, 13–15]. The advanced median age observed in this study further reflects the ongoing demographic shift toward an older patient population affected by these injuries [2, 21]. Notably, in the later treatment period, patients treated with THA ± ORIF were significantly older and exhibited higher comorbidity indices, suggesting an evolution in treatment algorithms toward arthroplasty-based strategies in more fragile patients. This is in line with contemporary concepts emphasizing individualized treatment decisions based on patient-related factors in addition to fracture morphology [6, 11]. Nevertheless, this observation reflects the intended evolution of the institutional treatment algorithm rather than random variation in patient selection. Consequently, differences between treatment groups should be interpreted within the context of indication-based treatment allocation. While the favourable outcomes observed after THA are encouraging, they cannot be interpreted as evidence of superiority of the procedure itself independent of the clinical decision process.

Compared to previous work from our institution that evaluated THA for acetabular fractures in patients aged ≥ 55 years, the present study extends these findings to a substantially older and more comorbid population [16]. Importantly, this development was an explicit objective of the redefined institutional SOP, which was implemented to standardize treatment allocation and facilitate the use of acute THA in elderly patients with fracture patterns and patient characteristics considered less favourable for joint-preserving fixation. By excluding the transitional implementation phase of the newly implemented SOP, potential confounding factors related to a learning curve with the new implant and treatment variability was minimized. This methodological approach strengthens the validity of the observed outcomes and suggests that the benefits of THA ± ORIF are reproducible under controlled clinical conditions and robust patient selection, while suggesting that the increased implementation of THA in the geriatric population is feasible and safe.

A key finding of this study is the significantly shorter time to mobilization observed in the THA ± ORIF group. Early mobilization is a critical determinant of outcome in geriatric trauma patients [22, 23]. The ability to allow early or immediate weight bearing after THA ± ORIF likely contributes to this advantage, as previously demonstrated in both, systematic reviews and comparative clinical studies [11, 12]. In contrast, ORIF often necessitates restricted weight bearing, which can be difficult to maintain in elderly patients and may delay rehabilitation [24]. The observed improvement in mobility at discharge in the THA ± ORIF cohort is therefore likely multifactorial, reflecting both earlier weight bearing and improved pain control.

Pain reduction represents another important benefit observed in the THA ± ORIF group. Patients undergoing THA ± ORIF reported significantly lower pain levels at discharge, which is consistent with previous findings suggesting that arthroplasty addresses both fracture-related instability and pre-existing degenerative joint disease [15]. Adequate pain control is known to facilitate postoperative rehabilitation and improve functional outcomes and may therefore have contributed to the superior mobilization observed in this cohort [25, 26].

The aforementioned functional advantages after THA were not associated with a significant difference in length of hospital stay between the treatment groups. This finding highlights the complex interplay of non-surgical factors influencing hospitalization in geriatric patients. Comorbidities, cognitive impairment, and organizational aspects of discharge planning have been shown to substantially affect length of stay, often outweighing the impact of the surgical procedure itself [27, 28].

More importantly, the overall complication rates did not differ significantly between treatment strategies. This finding is consistent with recent literature suggesting that THA in the setting of acetabular fractures does not necessarily result in higher complication rates when appropriately indicated [11]. Although arthroplasty in fracture settings has historically been associated with increased risks compared to elective procedures, the present results support the safety of THA ± ORIF in a carefully selected geriatric population [29]. Although the present study concerns acute traumatic acetabular fractures rather than intraoperative acetabular defects during primary THA, a recent systematic review of acetabular reconstruction strategies similarly emphasizes the importance of stable acetabular fixation and appropriate reconstruction to minimize implant-related complications and revision surgery [30]. It should be noted, however, that complication rates in elderly patients remain high irrespective of treatment modality, reflecting the intrinsic vulnerability of this population [9, 31].

The observed in-hospital mortality rate of 3.3% is comparable to previously reported rates in operatively treated patients, although slightly higher than figures reported in younger cohorts [32]. This difference is likely attributable to the higher age and comorbidity burden in the present study population. Furthermore, a subset of patients initially treated with ORIF required secondary conversion to THA. While the observed conversion rate of 8.6% is lower than the pooled rate reported in systematic reviews, it nevertheless underscores the risk of secondary procedures following joint-preserving surgery in elderly patients [6]. When interpreted in the context of the institutional SOP, this finding may reflect improved patient selection and a more differentiated allocation of patients to either ORIF or acute THA based on fracture morphology and patient-related factors. Together with the significantly faster mobilization and the absence of increased complication rates observed in the THA ± ORIF cohort, the comparatively low conversion rate supports the effectiveness of the SOP in identifying patients who are likely to benefit from primary arthroplasty and reducing the need for subsequent revision procedures.

The consistency of findings across treatment periods is of particular relevance. Despite treating older and more comorbid patients with THA ± ORIF in the later period, the advantages regarding mobilization and pain reduction persisted. This supports the robustness of the observed treatment effects and suggests that they may be attributable to the intervention and patient selection themselves rather than patient selection alone.

### Study strengths and limitations

This study provides detailed real-world data from a well-defined geriatric cohort treated at a high-volume Level 1 trauma center, with consistent surgical expertise and comprehensive clinical documentation. Stratification by treatment period and exclusion of the transitional phase enhance internal validity and allow for a more reliable assessment of treatment effects.

However, several limitations must be considered. First, the retrospective design introduces substantial potential for selection bias. Treatment allocation was not randomized but intentionally followed an evolving institutional treatment algorithm, whereby older and more medically frail patients were increasingly allocated to acute THA following implementation of the revised SOP. Consequently, this study is primarily an evaluation of a changing treatment strategy rather than a direct comparison of ORIF and THA. Accordingly, the observed associations are susceptible to confounding by indication [33]. Although baseline characteristics were reported separately by treatment period and procedure, residual confounding from measured and unmeasured patient factors cannot be eliminated. Therefore, causal inferences regarding the superiority of one surgical procedure over another should be avoided. In addition, residual confounding related to temporal changes in perioperative care cannot be excluded in the period-based analyses. The relatively small sample size may limit the statistical power to detect differences in infrequent outcomes such as complications and mortality. Consequently, the absence of statistically significant differences in these endpoints should not be definitively interpreted as evidence of equivalence between treatment strategies. Furthermore, outcomes were limited to the inpatient period. Consequently, the present findings are restricted to early postoperative recovery and cannot be extrapolated to long-term functional outcomes, implant survival, revision risk, or quality of life. Finally, the single-centre design may limit external validity.

## Conclusion

In conclusion, the implementation of a structured treatment algorithm incorporating THA ± ORIF for selected geriatric patients with displaced acetabular fractures was associated with earlier postoperative mobilization, reduced pain levels, and improved short-term functional outcomes without an increased risk of complications. Notably, these advantages were observed despite the treatment of older and more medically comorbid patients following implementation and refinement of the institutional SOP. Together with the comparatively low rate of secondary conversion from ORIF to THA, these findings suggest that differentiated treatment allocation facilitates the safe and effective use of acute THA in carefully selected elderly patients. The consistency of outcomes after the establishment of the SOP further supports its applicability in routine clinical practice. Nevertheless, treatment decisions should remain individualized and based on fracture morphology, comorbidity burden, bone quality, and pre-injury functional status. Prospective multicenter studies with larger cohorts and long-term follow-up are warranted to further validate these findings and establish evidence-based treatment recommendations.

## Supplementary Information

Below is the link to the electronic supplementary material.

Supplementary Material 1

## Data Availability

The data collected will be stored securely at our institute for 10 years. During this period, they are still available upon request. After 10 years, the data will be deleted, however, all the datasets analyzed or generated during this study will be available from the corresponding author upon reasonable request.
