# Supplementary material for: Management and outcome of displaced acetabular fractures in the oldest-old: a retrospective analysis of orif and acute total hip arthroplasty
Source: Arch Orthop Trauma Surg. 2026 Sep 18;146(1):340. doi: 10.1007/s00402-026-06511-0 (PMC13588819; doi:10.1007/s00402-026-06511-0)
Supplement: Supplementary file 1 [file 402_2026_6511_MOESM1_ESM.docx]

**Supplementary Figure S1.** Institutional treatment algorithm for the management of geriatric acetabular fractures implemented at our institution in April 2019. The algorithm is adapted from our previously published institutional treatment protocol [1].

References

1. Rashed M, Hildebrand F, Hofmann U, Horst K, Hürtgen B, Bolierakis E, Berk T, Alabdulrahman H. Acute total hip replacement of acetabular fractures with cementless modular revision cups in patients older than 55 years: a retrospective cohort study. Eur J Trauma Emerg Surg. 2025;51:362.
